# Arabinogalactan Alleviates Lipopolysaccharide-Induced Intestinal Epithelial Barrier Damage through Adenosine Monophosphate-Activated Protein Kinase/Silent Information Regulator 1/Nuclear Factor Kappa-B Signaling Pathways in Caco-2 Cells

**DOI:** 10.3390/ijms242015337

**Published:** 2023-10-19

**Authors:** Jiachen Zheng, Shaoying Gong, Jianchun Han

**Affiliations:** 1 College of Food Science, Northeast Agricultural University, Harbin 150030, China; zhengjiachen0422@126.com; 2 Heilongjiang Green Food Science Research Institute, Northeast Agricultural University, Harbin 150030, China

**Keywords:** arabinogalactan, intestinal epithelial barrier, tight junction proteins, intracellular calcium, oxidative stress, inflammatory, AMPK/SIRT1/NF-κB signaling pathway

## Abstract

Intestinal epithelial barrier (IEB) damage is an important aspect in inflammatory bowel disease (IBD). The objective of this study was to explore the protective effects and mechanisms of arabinogalactan (AG) on lipopolysaccharide (LPS)-stimulated IEB dysfunction. The results show that AG (1, 2, and 5 mg/mL) mitigated 100 μg/mL LPS-stimulated IEB dysfunction through increasing transepithelial electrical resistance (TEER), reducing fluorescein isothiocyanate (FITC)–dextran (4 kDa) flux, and up-regulating the protein and mRNA expression of tight junction (TJ) proteins (Claudin-1, Zonula occludens-1 (ZO-1) and Occludin). In addition, AG ameliorated LPS-stimulated IEB dysfunction by reducing interleukin-6 (IL-6), tumor necrosis factor-α (TNF-α), and IL-1β levels, decreasing the reactive oxygen species (ROS) level, increasing superoxide dismutase (SOD) activity, increasing the glutathione (GSH) level, and decreasing the levels of malondialdehyde (MDA) and intracellular calcium ([Ca^2+^]_i_). Furthermore, 2 mg/mL AG up-regulated the expression of silent information regulator 1 (SIRT1), the phosphorylated adenosine monophosphate-activated protein kinase (AMPK), and peroxisome proliferator-activated receptor gamma coactivator (PGC)-1α and inhibited the phosphorylation of nuclear factor kappa-B (NF-κB) and the inhibitor of NF-κBα (IκBα). Therefore, AG could maintain IEB integrity by activating AMPK/SIRT1 and inhibiting the NF-κB signaling pathway. In conclusion, AG can regulate the AMPK/SIRT1/NF-κB signaling pathway to reduce inflammation and oxidative stress, thus alleviating LPS-stimulated IEB damage.

## 1. Introduction

Inflammatory bowel disease (IBD), a chronic inflammatory disease, commonly presents with diarrhea and weight loss, and in serious cases, it leads to cancer [1,2]. The incidence of IBD has increased in recent years due to changes in dietary habits and environmental factors [3]. According to recent statistics, there are over 6.8 million people who are diagnosed with IBD globally [4]. IBD is characterized by damage to the intestinal epithelial barrier’s (IEB) integrity [5]. Studies have reported that oxidative stress and inflammatory reactions can lead to the destruction of the IEB through damage to tight junctions (TJs) [6,7,8,9]. Reactive oxygen species (ROS), cell byproducts, bring about oxidative stress and cell damage [10]. An increase in ROS content results in calcium release from the endoplasmic reticulum to the cytoplasm, thus increasing intracellular calcium ([Ca^2+^]_i_) content [11]. [Ca^2+^]_i_ is necessary for assembling TJs and maintaining the integrity of TJs [12]. When harmful substances invade, the integrity of TJs is destroyed, which may result in over-activation of the inflammation response [13,14] Therefore, maintaining IEB integrity might be an efficacious method of mitigating IBD.

The pathogenesis of IBD has not been fully elucidated and may be related to immune response disorders, epithelial barrier defects, genetic susceptibility, and external environmental stimuli [5,15]. Current clinical treatment methods for IBD mainly include using biological agents, salicylates, and immunomodulators [5,16]. However, due to their side effects, there is an ongoing search for alternatives for the treatment of IBD, such as traditional Chinese medicines, polyphenols, polysaccharides, dietary fiber, and other natural compounds [17,18]. Dietary fiber as a prebiotic for gut health has the advantage of low toxicity and fewer side effects and can be split into soluble and insoluble dietary fiber [19,20]. Arabinogalactan (AG) is a highly safe, soluble dietary fiber and can be used as a food additive [20]; it is abundant in coniferous trees, for instance, *Mangifer aindica* L., *Astralagus gummifer*, and larch wood [21]. AG possesses immuno-enhancing [22], anti-cancer [23], anti-inflammatory [24], and antioxidant activities [25]. In addition, AG has the ability to alleviate cisplatin-stimulated intestinal injury [26]; however, the mechanism of AG’s effect on IEB function is still not fully understood.

Lipopolysaccharides (LPSs), a common inflammatory mediator, can damage the IEB through inhibiting TJ protein expression, resulting in chronic diseases like IBD [14,27]. Therefore, in this research, we used Caco-2 monolayers to assess the beneficial impact of AG and its possible role in LPS-stimulated IEB damage. Our results indicate that AG could alleviate IEB injury by regulating the AMPK/SIRT1/NF-κB signaling pathway. This study provides a theoretical basis for the use of natural products to alleviate IBD and expand the utilization of AG.

## 2. Results

### 2.1. AG Mitigated LPS-Induced Cytotoxicity

**Figure 1:**
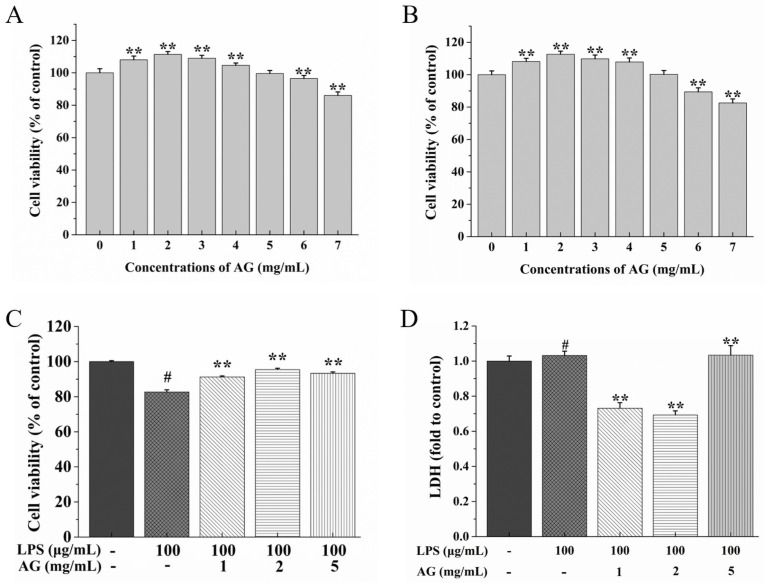
Effects of arabinogalactan (AG) on Caco-2 cell viability after (**A**) 24 h and (**B**) 48 h of treatment. (**C**) Effects of co-treatment with AG and LPS on Caco-2 cell viability. (**D**) Effects of co-treatment with AG and LPS on lactate dehydrogenase (LDH) leakage. Data represent the means of three independent experiments. (**A**,**B**) ** indicates *p* < 0.01 relative to the control group, (**C**,**D**) # and ** indicate *p* < 0.01 relative to the control group and LPS group, respectively.

To evaluate whether AG is toxic to Caco-2 cells, CCK-8 was used to measure the impact of AG (1–7 mg/mL) on cell activity. In comparison to the control cells, treatment with AG for 24 h (Figure 1A) or 48 h (Figure 1B) had no toxic effects on Caco-2 cells with a 0–5 mg/mL addition. Then, we assessed the effect of co-treatment with AG and LPS on cytotoxicity (Figure 1C). LPS reduced cell viability, while AG increased cell viability in a concentration-dependent manner. Thus, LPS (100 μg/mL) and AG at concentrations of 1, 2, and 5 mg/mL were used in subsequent experiments.

The impact of AG on LPS-stimulated cell cytotoxicity could also be evaluated by considering lactate dehydrogenase (LDH) leakage. As shown in Figure 1D, when the cells were treated with LPS, LDH leakage increased, as expected (*p* < 0.01). The cells treated with AG attenuated this increase, and with the increase in the AG concentration, the level of LDH was lowered.

### 2.2. AG Protected Intestinal Epithelial from LPS Stimulation in Caco-2 Monolayer

**Figure 2:**
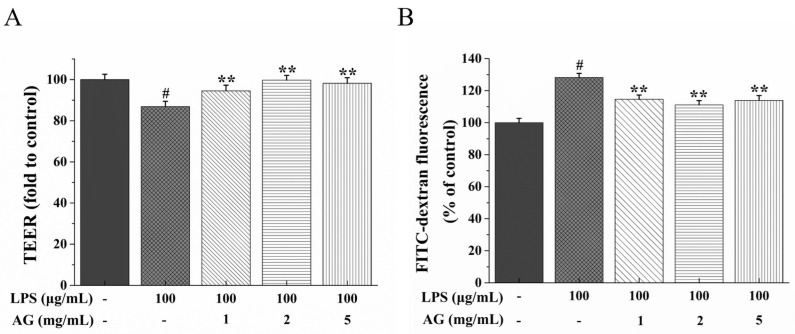
Effects of AG on (**A**) transepithelial electrical resistance (TEER) and (**B**) FITC-dextran fluorescence value in LPS-stimulated Caco-2 cell monolayers after treatment for 24 h. Data represent the means of three independent experiments. # and ** indicate *p* < 0.01 relative to the control group and LPS group, respectively.

Transepithelial electrical resistance (TEER) and fluorescein isothiocyanate (FITC)–dextran (4 kDa) flux were used as the indicators to estimate the integrity of Caco-2 monolayers. As shown in Figure 2A, relative to the control monolayer, LPS significantly reduced TEER (*p* < 0.01), while treatment with AG significantly improved this downward trend. The fluorescence value of FITC-dextran increased after LPS exposure; however, with increasing concentrations of AG (1, 2, and 5 mg/mL), the fluorescence intensity gradually decreased (Figure 2B).

### 2.3. Effect of AG on TJs in a Caco-2 Cell Monolayers

**Figure 3:**
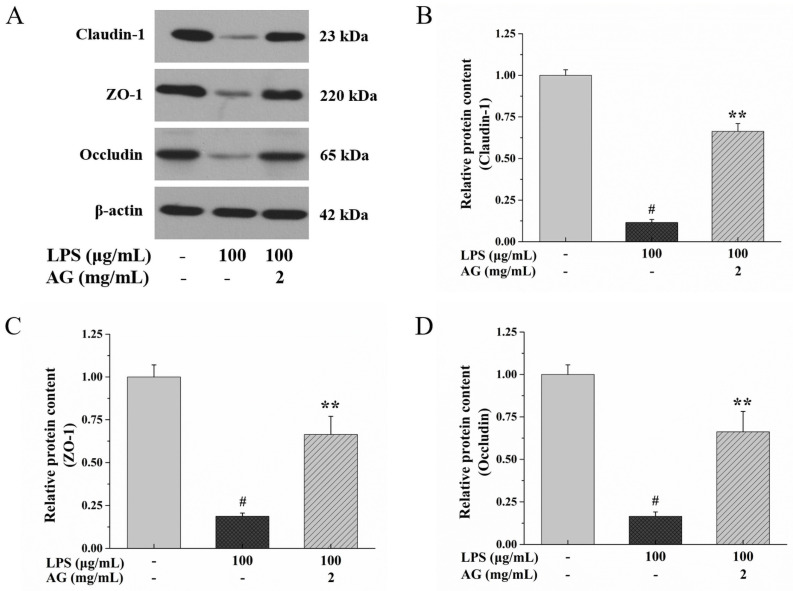
Effects of AG on tight junction (TJ) protein expression in LPS-stimulated Caco-2 cell monolayers. (**A**) Western blotting images of claudin-1, ZO-1, and occludin protein expression. Relative protein expression levels of (**B**) claudin-1, (**C**) ZO-1, and (**D**) occludin. Data represent the means of three independent experiments. # and ** indicate *p* < 0.01 relative to the control group and LPS group, respectively.

**Figure 4:**
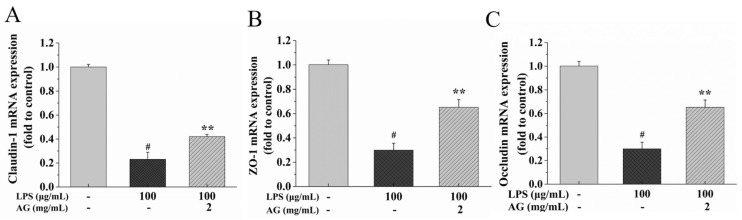
Effects of AG on the relative mRNA expressions of tight junction (TJ) proteins: (**A**) claudin-1, (**B**) ZO-1, and (**C**) occludin in LPS-stimulated Caco-2 cell monolayers. Data represent the means of three independent experiments. # and ** indicate *p* < 0.01 relative to the control group and LPS group, respectively.

TJs are part of the IEB and are interrelated to intestinal permeability. Therefore, we evaluated TJ protein (ZO-1, claudin-1, and occludin) expression after LPS and AG treatment through Western blotting and RT-PCR experiments. In comparison to control group, LPS conspicuously decreased the protein and mRNA expressions of ZO-1, claudin-1, and occludin, while AG co-treatment alleviated this down-regulation (Figure 3 and Figure 4).

### 2.4. Effect of AG on Relieving LPS-Stimulated Inflammation Level

**Figure 5:**
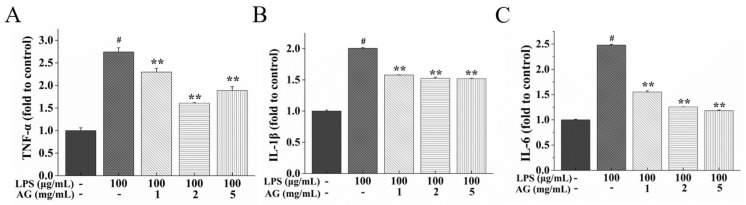
Effects of AG on pro-inflammatory cytokines: (**A**) TNF-α, (**B**) IL-1β, and (**C**) IL-6 in LPS-stimulated Caco-2 cell monolayers. Data represent the means of three independent experiments. # and ** indicate *p* < 0.01 relative to the control group and LPS group, respectively.

In comparison to the control group, the levels of interleukin (IL)-1β, IL-6, and tumor necrosis factor-α (TNF-α) in the LPS-treated cell monolayer increased, as expected (Figure 5). AG, when co-cultured with LPS, significantly eliminated this increase (*p* < 0.01). An AG concentration of 2 mg/mL showed the highest anti-inflammatory ability.

### 2.5. Effect of AG on Oxidative Stress

**Figure 6:**
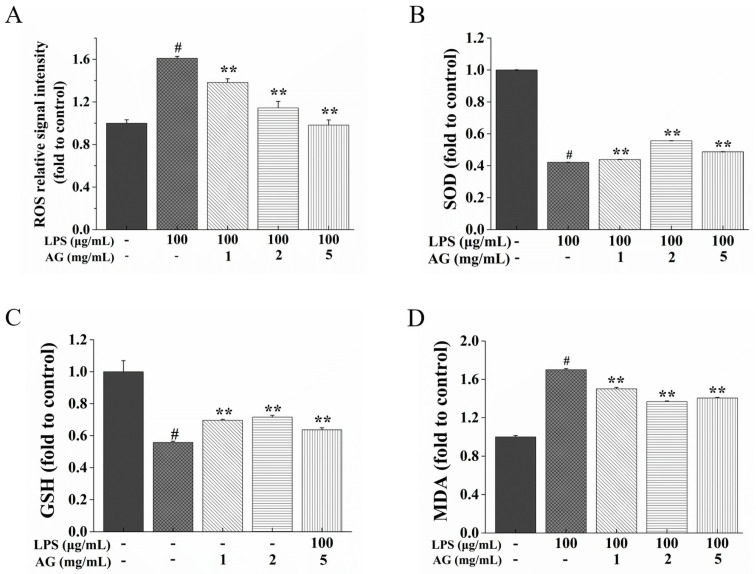
Effects of AG on (**A**) ROS signal intensity, (**B**) SOD activity, (**C**) GSH content, and (**D**) MDA content in LPS-stimulated Caco-2 cells. Data represent the means of three independent experiments. # and ** indicate *p* < 0.01 relative to the control group and LPS group, respectively.

After exposure to LPS, the ROS level showed an eminent increase (*p* < 0.01) (Figure 6A). However, AG (1, 2, and 5 mg/mL) suppressed this increase (*p* < 0.01). Superoxide dismutase (SOD) activity and glutathione (GSH) content were attenuated, while the malondialdehyde (MDA) content in cells increased with LPS treatment (Figure 6B–D). At the same time, after AG treatment, SOD activity and GSH content increased, while the content of MDA decreased.

### 2.6. Effect of AG on [Ca^2+^]_i_

**Figure 7:**
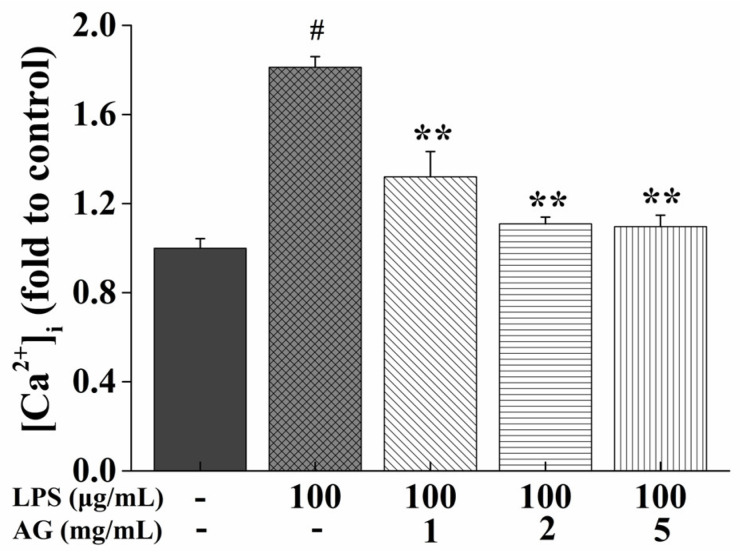
Effects of AG on intracellular calcium ([Ca^2+^]_i_) content in LPS-stimulated Caco-2 cells. Data represent the means of three independent experiments. # and ** indicate *p* < 0.01 relative to the control group and LPS group, respectively.

In our study, we measured the level of [Ca^2+^]_i_. As shown in Figure 7, the cells exposed to LPS had increased [Ca^2+^]_i_ levels compared to the control cells. However, co-treatment with AG and LPS inhibited this increase.

### 2.7. Effect of AG on the AMPK/SIRT1 and NF-κB Signaling Pathways

**Figure 8:**
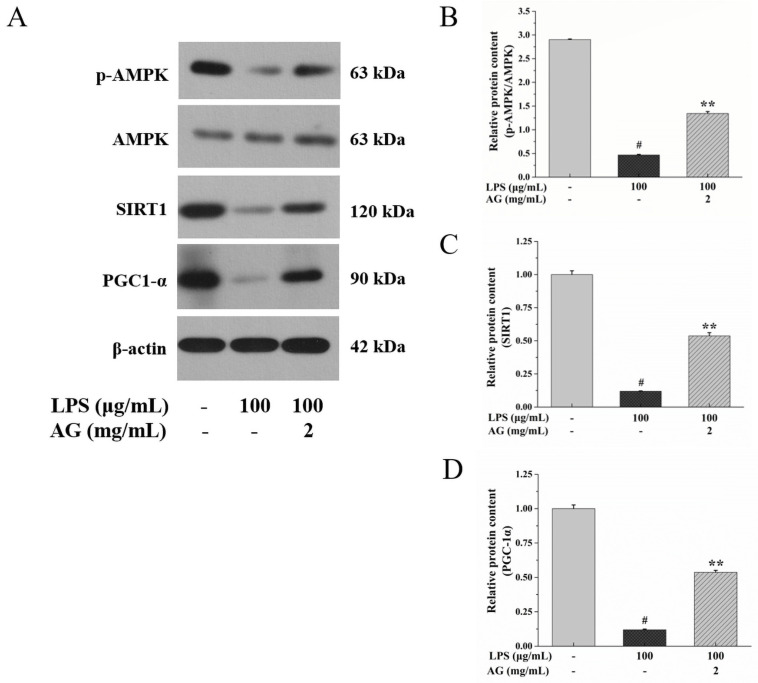
Effects of AG on LPS-stimulated Caco-2 cells. (**A**) Western blotting images of p-AMPK, AMPK, SIRT1, and PGC1α protein expression. Relative protein expression of (**B**) the ratio of p-AMPK/AMPK, (**C**) SIRT1, and (**D**) PGC1α. Data represent the means of three independent experiments. # and ** indicate *p* < 0.01 relative to control group and LPS group, respectively.

**Figure 9:**
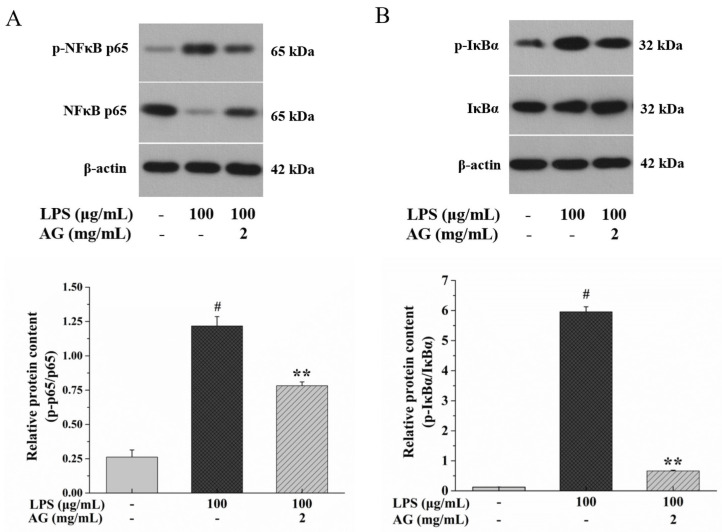
Effects of AG on (**A**) Western blotting images of p-NF-κB p65 and NF-κB p65 and the ratio of p-NF-κB p65/NF-κB p65. (**B**) Western blotting images of p-IκBα and IκBα and the ratio of p-IκBα/IκBα in LPS-induced Caco-2 cells. Data represent the means of three independent experiments. # and ** indicate *p* < 0.01 relative to the control group and LPS group, respectively.

To study the effect of AG on the AMPK/SIRT1 and NF-κB pathways, we measured the expression of AMPK, p-AMPK, SIRT1, PGC-1α, NF-κB p65, p-NF-κB p65, IκBα, and p-IκBα through Western blotting. In comparison to the control group, after exposure to LPS, the expression of p-AMPK, SIRT1, and PGC-1α decreased (Figure 8), while p-NF-κB p65 and p-IκBα protein expression increased, as expected (Figure 9). When the cells were exposed to 100 μg/mL LPS and 2 mg/mL AG, the expression of p-AMPK, SIRT1, and PGC-1α increased; however, the expression of p-NF-κB p65 and p-IκBα proteins was inhibited compared to the cells exposed to LPS alone. These results show that AG alleviated intestinal epithelial barrier damage through activating AMPK/SIRT1 and inhibiting the NF-κB signaling pathway.

## 3. Discussion

Cell activity and LDH release can be used to evaluate cell damage. When cells are stimulated, LDH, as a key indicator of cell damage, is released to the outside of cells [28,29]. Thus, we detected the impact of AG on LPS-induced cell damage by assessing cell viability and LDH leakage. A previous study showed that the handroanthus heptaphyllus polysaccharide fraction (HHSF), which consists of a type II arabinogalactan, had no toxicity to cells when the concentration was 0, 10, 100, or 1000 μg/mL [30]. Similarly, our study showed that AG was nontoxic to cells at concentrations of 0–5 mg/mL (Figure 1A,B). Xiong et al. [29] found that LDH leakage in an LPS-treated group was conspicuously higher than that in the control group. Furthermore, AG could reduce the increase in LDH leakage in cisplatin-induced intestinal injury and thus increase cell viability [26]. Consistent with the results of previous studies, our study showed that Caco-2 cells treated with LPS showed an increase in LDH leakage, while co-treatment with AG attenuated this trend (Figure 1D). These results suggest that AG could alleviate cell damage caused by LPS in Caco-2 cells.

The integrity and permeability of the IEB can be assessed with TEER and FITC-dextran [6,7,12,28]. A lower TEER indicates that a higher current passes through the damaged cells and the TJs [7,28,31]. Dextran is an indigestible polysaccharide with molecular sizes ranging from 3-kDa to 2000-kDa [32]. Among them, 4-kDa FITC-dextran is commonly used to measure intestinal permeability [33]. When the IEB is impaired, 4-kDa FITC-dextran migrates to the intestinal serosa and then enters the systemic circulation [33]. Gong et al. [7] found that the TEER value decreased and FITC-D4 flux increased when Caco-2 cell monolayers were exposed to LPS. Consistent with previous research findings, as shown in Figure 2A,B, with LPS treatment, the TEER value decreased and the flux of FITC increased, while AG improved LPS-stimulated IEB damage. The increase in the TEER value and decrease in FITC-dextran flux might be due to the down-regulation of TJ proteins [34]. TJs are made up of junctional adhesion molecules (JAMs), ZO-1, occludin, and claudins [14]. The regulation of TJ proteins promotes the integrity of barriers formed to protect against harmful antigens and thus prevent intestinal inflammation [13,34]. ZO-1, occludin, and claudin-1 are important proteins in maintaining the barrier, limiting and adjusting TJ paracellular permeability [14,34]. Among these proteins, occludin, a tight junction model protein, is essential for regulating paracellular permeability in epithelial monolayers [35]. LPS can down-regulate the mRNA and protein expressions of TJ proteins, disrupt TJ continuity, and damage the integrity of the IEB [7]. AG from *Lycium barbarum* could partially restore the IEB integrity damaged by dextran sulfate sodium by up-regulating TJ protein expression at the gene and protein levels [36]. Our research showed that LPS down-regulated TJ protein expression, while AG up-regulated the TJ protein expression (Figure 3 and Figure 4). The results show that AG can alleviate LPS-stimulated IEB damage.

TJ protein expression might be regulated by pro-inflammatory cytokines [13,14]. One study indicated that LPS could impair IEB integrity by triggering pro-inflammatory cytokine release [7]. Consistent with previous research findings, LPS treatment was found to induce the release of inflammatory factors, while co-treatment with AG suppressed this release (Figure 5). Our study showed that AG could alleviate LPS-stimulated intestinal damage through reducing inflammatory factor secretion.

In addition, TJ protein expression might be affected by oxidative stress [37]. When the production of ROS is higher than the antioxidant capacity, it leads to oxidative stress and damage to proteins and cells, eventually leading to IEB damage [15]. In our study, with LPS treatment, the production of ROS was increased, while ROS content was decreased with AG treatment (Figure 6A). Cells have enzymatic and non-enzymatic defense systems to protect against ROS; among these, SOD and GSH are natural antioxidant enzymes [38]. SOD can transform superoxides into peroxides [38]. GSH can remain in the reduced form to capture free radicals and ROS, thereby preventing the occurrence of oxidative stress [39]. Furthermore, free radicals can react with polyunsaturated fatty acids, causing lipid peroxidation and producing lipid peroxides, such as MDA [38]. It has been proven that LPS can induce intestinal oxidative damage by reducing SOD activity and GSH content and increasing MDA content [8]. Sun et al. [25] found that AG from black soybean increased SOD activity and GSH levels in mice with carbon tetrachloride-stimulated acute liver injury. This study showed that exposure to LPS decreased SOD activity and the GSH level and increased MDA content (Figure 6B–D), while exposure to AG suppressed these changes. Thus, we found that AG alleviates LPS-stimulated IEB damage via inhibiting oxidative stress.

Calcium is an essential and crucial second messenger when cells are stimulated to generate cytoplasmic Ca^2+^ signals, which are involved in secondary metabolism [40]. Ca^2+^ signal transduction is beneficial for the LPS-induced immune response [41]. LPS exposure led to the disturbance of [Ca^2+^]_i_ homeostasis and increased [Ca^2+^]_i_ concentration, thereby increasing the ROS levels and causing oxidative stress [42]. As shown in Figure 7, our study showed that LPS increased [Ca^2+^]_i_ content, while AG attenuated this increase.

It has been reported that [Ca^2+^]_i_ can activate AMPK [43,44]. Activated AMPK, a serine/threonine kinase, can enhance the integrity via promoting the assembly of TJs [43,45]. In addition, activated AMPK is a critical regulator that inhibits inflammation and oxidative stress by regulating the SIRT1 signaling pathway and is related to the etiology of IBD [16,46]. SIRT1 is a nicotinamide adenosine dinucleotide^+^-dependent protein deacetylase [37,46]. SIRT1 can adjust PGC-1α expression to decrease oxidative stress, protect the IEB, and restrain inflammatory factor secretion by inhibiting the NF-κB pathway [16,37], which is consistent with our results. Hwang et al. [34] found that LPS down-regulates AMPK and SIRT1, up-regulates NF-κB expression, and promotes the phosphorylation of NF-κB and IκBα, which is similar to our results. At the same time, AG activated AMPK/SIRT1 (Figure 8) and inhibited the NF-κB (Figure 9) signaling pathway to alleviate the intestinal injury caused by LPS.

## 4. Materials and Methods

### 4.1. Materials

LPS (obtained from *Escherichia coli O111:B4*) (Cas No. SAB4200878) and AG (≥84% HPLC) (Cas No. 9036-66-2) from larch wood was purchased from Sigma (Saint Louis, MO, USA). FITC-dextran (4 kDa) and penicillin/streptomycin (P/B) were obtained from Sigma. Fluo-4/AM and LDH, the Cell Counting Kit (CCK)-8, ROS, SOD, MDA, GSH, IL-6, IL-1β, TNF-α, and BCA protein assay kits were purchased from Beyotime Biotechnology (Shanghai, China).

### 4.2. Cell Culture

Caco-2 cells were obtained from the Cell Bank of the Chinese Academy of Sciences (Shanghai, China). Caco-2 cells were cultured with Dulbecco’s modified Eagle’s medium (HyClone, Logan, UT, USA) along with 10% fetal bovine serum (HyClone) and 1% P/B. Cells were cultivated at 37 °C in a 5% CO_2_ incubator. After reaching 90% confluence, cells were digested with 0.25% trypsin–ethylenediaminetetraacetic acid (HyClone) and passed through the flask.

### 4.3. Cell Viability Assay

LPS and AG were diluted in serum-free medium to obtain stock solutions with concentrations of 100 μg/mL and 50 mg/mL, respectively. Then, the LPS and AG stock solutions were both stored at −20 °C.

CCK-8 was utilized to measure cell activity using the method of Yuan et al. [28]. The cells (8 × 10^5^ cells/mL) were inoculated into 96-well plates for 24 h and then cultured with AG (0, 1, 2, 3, 4, 5, 6, or 7 mg/mL) for 24 h or 48 h or AG (0, 1, 2, and 5 mg/mL) with LPS for 24 h. After incubating, CCK-8 was added to the adherent cells and incubated for 30 min in the incubator. Then, the absorbance was measured at 450 nm using a microplate reader (Infinite 200 PRO, Tecan, Grodig, Austria).

### 4.4. LDH Activity Assay

LDH activity was detected using the experimental method of Gu et al. [47]. The cells were seeded into 6-well plates for 24 h. Next, the cells were exposure to the vehicle (set as control) or LPS with AG (0, 1, 2, or 5 mg/mL). After incubation for 24 h, the LDH activity in the supernatants was determined using an LDH assay kit. The absorbance was measured using a microplate reader at 450 nm, and the final value is presented as a ratio compared to control cells.

### 4.5. TEER Assay

The cells (at a density of 10^5^ cells/mL) were inoculated into a 12-well Transwell insert (Corning, Kennebunk, ME, USA). The medium was replaced every day from day one to day seven and changed every day after the first week. The TEER was measured using a Millicell-ERS Voltammeter (Millipore, Bedford, MA, USA). After a TEER above 400 Ω cm^2^ was attained, the monolayers were exposed to the vehicle or LPS with AG (0, 1, 2, or 5 mg/mL) [6]. After 24 h, the TEER was measured again using a Millicell-ERS Voltammeter, and the value of the TEER is expressed as a percentage in comparison to control cells.

### 4.6. Permeability Assay

The permeability was evaluated using 4 kDa FITC-dextran using the method of Fu et al. [12]. After the TEER exceeded 400 Ω cm^2^, the monolayers were exposed to the vehicle or LPS with AG (0, 1, 2, and 5 mg/mL) for 24 h. The cell monolayers were treated with 1 mg/mL FITC-dextran (dissolved by Hank’s balanced salt solution (HBSS)). One hundred microliters of basolateral solution was used to detect the fluorescence intensity using a fluorescence microplate reader with an excitation wavelength of 490 nm and an emission wavelength of 520 nm. The result is expressed as a percentage in comparison to control cells.

### 4.7. Enzyme-Linked Assay

TNF-α, IL-1β, and IL-6 levels were measured using the experimental method of Gong et al. [7]. After the TEER exceeded 400 Ω cm^2^, the monolayers were treated with the vehicle or LPS with AG (0, 1, 2, or 5 mg/mL) for 24 h. Then, the supernatant in the apical side was taken and the levels were measured with ELISA. The value is presented as a ratio compared to control cells.

### 4.8. Measurement of ROS Levels

ROS content was evaluated using a DCFH-DA fluorescent probe referring to the experimental method of Kim et al. [14]. Cells (10^5^ cells/well, 100 μL) were cultured in a 96-well plate for 24 h. Then, the cells were exposed to the vehicle (control) or 100 μg/mL LPS with AG (0, 1, 2, or 5 mg/mL). After 24 h, 10 μM DCFH-DA was added to the adherent cells for 30 min. The fluorescence value was read using a microplate reader with an excitation wavelength of 488 nm and emission wavelength of 525 nm. The results are presented as a ratio in comparison to the control cells.

### 4.9. Measurement of SOD Activity, and GSH and MDA Content in Caco-2 Cells

The SOD activity and the GSH and MDA contents measurements were used to reflect oxidative stress [28]. The cells (10^5^ cells/well, 2 mL) were seeded into 6-well plates (Corning) for 24 h, and then exposed to vehicle or LPS with AG (0, 1, 2 or 5 mg/mL) for 24 h. SOD activity and GSH and MDA content were measured according to the instructions. The results are presented as the ratio compared to control cells.

### 4.10. Assay of [Ca^2+^]_i_

Fluo-4/AM was used to determine [Ca^2+^]_i_ content [9]. The cells (10^5^ cells per well) were seeded into 12-well plates (Corning). After 24 h, cells were exposed to vehicle, or LPS with AG (0, 1, 2 or 5 mg/mL) for 24 h. Next, the cells were exposed to 5 μmol/L Fluo-4/AM (in HBSS) for 30 min. The fluorescence intensity was determined using a fluorescence microplate reader with an excitation wavelength of 494 nm and an emission wavelength of 516 nm. The results are shown as the ratio in comparison to the control cells.

### 4.11. Assay of Western Blot

Western blotting was performed according to a previously published method [48]. After culturing with vehicle (control), LPS, or LPS with AG (2 mg/mL) for 24 h, cells were exposed to RIPA buffer, lysed on ice for about 30 min, and centrifuged for 20 min at a speed of 12,000× *g*. The total protein was assayed with a BCA Assay Kit. Proteins were utilized for 10% sodium dodecyl sulfate polyacrylamide gel electrophoresis and then transferred to PVDF membranes and blocked for 2 h. Next, the membranes were incubated overnight with a primary antibody: ZO-1 (1:1000, Wanlei Life Sciences Co., Ltd., Shenyang, China), anti-occludin (1:1000, Wanlei Life Sciences), anti-claudin-1 (1:2000, Wanlei Life Sciences) and β-actin (1:1000, Wanlei Life Sciences), anti-SIRT-1 polyclonal antibody (1:500, Wanlei Life Sciences), anti-AMPK antibody (1:500, Wanlei Life Sciences), anti-p-AMPK antibody (1:500, Wanlei Life Sciences), anti-PGC1α antibody (1:750, Wanlei Life Sciences), anti-NF-κB p65 antibody (1:1000, Wanlei Life Sciences); anti-NF-κB p-p65 antibody (1:1000, Wanlei Life Sciences); anti- IκBα antibody (1:500, Wanlei Life Sciences); and anti-p-IκBα antibody (1:1000, Wanlei Life Sciences). Then, the membranes were incubated with secondary antibody (1:3000, Wanlei Life Sciences) for 1 h in a 37 °C incubator. The protein bands were visualized with a ChemiDoc Imaging System. β-actin was used to normalize the protein levels, and the results are represented as a proportion in comparison to control cells.

### 4.12. Assay of RT-PCR

The total RNA was extracted using TRIzol reagent and analyzed with a spectrophotometer (NanoDrop 2000, Thermo Scientific, Waltham, MA, USA). After that, 500 ng of total RNA was used to synthesize cDNA via reverse transcription with the PrimeScript™ RT reagent kit (TaKaRa Bio Ltd., Shiga, Japan). Then, RT-PCR was performed with TB Green™ Premix Ex Taq ™ (TaKaRa) using 1 μL of cDNA template. The amplification program was 95 °C for 30 s, 45 cycles of 95 °C for 5 s, and 60 °C for 30 s [7]. Glyceraldehyde-3-phosphate dehydrogenase (GAPDH) was used as the internal control gene. Primer sequences are shown in Table S1.

### 4.13. Statistical Analysis

The results were analyzed using ANOVA and Duncan analysis in SPSS 24.0, and the value is shown as the mean value ± standard error (SE). *p* < 0.05 was deemed to signify remarkable differences in different groups. Three replicates were performed for each experiment.

## 5. Conclusions

In conclusion, our study showed that AG inhibited oxidative stress and inflammation via activating AMPK/SIRT1 and inhibiting the NF-κB signaling pathway, thereby promoting TJ protein expression and alleviating LPS-stimulated IEB damage. This study broadens our understanding of AG’s function and provides a theoretical basis for the development and utilization of AG as a functional food additive to alleviate IBD. Our study mainly involved in vitro research and lacks in vivo validation. In the future, we will conduct in-depth research in vivo and continue to explore whether other protein or gene targets play a role in preventing intestinal barrier damage.

## Data Availability

The data provided in this study are available on request from the corresponding author.

## Supplementary Materials

The following supporting information can be downloaded at: https://www.mdpi.com/article/10.3390/ijms242015337/s1.
