# Supplementary material for: Arabinogalactan Alleviates Lipopolysaccharide-Induced Intestinal Epithelial Barrier Damage through Adenosine Monophosphate-Activated Protein Kinase/Silent Information Regulator 1/Nuclear Factor Kappa-B Signaling Pathways in Caco-2 Cells
Source: Int J Mol Sci. 2023 Oct 19;24(20):15337. doi: 10.3390/ijms242015337 (PMC10607795; doi:10.3390/ijms242015337)
Supplement: Supplementary file 1 [file ijms-24-15337-s001.zip › ijms-2608187-supplementary.pdf]

Table S1 Sequence of target gene primers.

Genes	Forward primer	Reverse primer
Claduin-1	GAAGTGCTTGGAAGACGATG	GAGCCTGACCAAATTCGTAC
ZO-1	TCACGCAGTTACGAGCAAGT	TGAAGGTATCAGCGGAGGGA
Occludin	TCAGGGAATATCCACCTATCACTTCAG	CATCAGCAGCAGCCATGTACTCTTCAC
GAPDH	GTCTCCTCTGACTTCAACAGCG	ACCACCCTGTTGCTGTAGCCAA
